# Oxidative Stress Modulation and ROS-Mediated Toxicity in Cancer: A Review on *In Vitro* Models for Plant-Derived Compounds

**DOI:** 10.1155/2017/4586068

**Published:** 2017-10-24

**Authors:** María José Vallejo, Lizeth Salazar, Marcelo Grijalva

**Affiliations:** ^1^Departamento de Ciencias de la Vida, Universidad de las Fuerzas Armadas ESPE, Avenida General Rumiñahui S/N, P.O. Box 171-5-231B, Sangolquí, Ecuador; ^2^Centro de Nanociencia y Nanotecnología, Universidad de las Fuerzas Armadas ESPE, Avenida General Rumiñahui S/N, P.O. Box 171-5-231B, Sangolquí, Ecuador

## Abstract

Medicinal and aromatic plants (MAPs) are known and have been long in use for a variety of health and cosmetics applications. Potential pharmacological usages that take advantage of bioactive plant-derived compounds' antimicrobial, antifungal, anti-inflammatory, and antioxidant properties are being developed and many new ones explored. Some phytochemicals could trigger ROS-mediated cytotoxicity and apoptosis in cancer cells. A lot of effort has been put into investigating novel active constituents for cancer therapeutics. While other plant-derived compounds might enhance antioxidant defenses by either radical scavenging or stimulation of intracellular antioxidant enzymes, the generation of reactive oxygen species (ROS) leading to oxidative stress is one of the strategies that may show effective in damaging cancer cells. The biochemical pathways involved in plant-derived bioactive compounds' properties are complex, and *in vitro* platforms have been useful for a comprehensive understanding of the mechanism of action of these potential anticancer drugs. The present review aims at compiling the findings of particularly interesting studies that use cancer cell line models for assessment of antioxidant and oxidative stress modulation properties of plant-derived bioactive compounds.

## 1. Introduction

Cell culture techniques allow investigators to look *in vitro* into the effect of plant compounds under controlled conditions that ensure consistency and reproducibility of results. Nevertheless, interactions with other cell types are not usually considered in cell culture assays, so that the *in vivo* environment might not be fully mimicked. As a consequence, experimental design should be done carefully, with appropriate controls included in every assay [1].

There are several strategies for oxidative stress modulation such as ionizing radiation [2] and platinum coordination complexes [3], which are widely used in cancer treatment that significantly increases ROS expression levels. Furthermore, radiation exposure generates high production of NADPH oxidase, causing persistent OS [4]. Other interesting examples are anthracyclines, which induce production of superoxide radicals [5] and arsenic trioxide drugs that stimulate ROS production within the mitochondria via p53 activation [6]. In recent years, however, there is a growing interest in medicinal and aromatic plants (MAPs) and their antioxidant and oxidative stress modulation properties. This field of research looks particularly interesting for cancer therapeutics. A sizeable part of this research is made on cancer cell models. In the next sections, an overview of *in vitro* assessment of medicinal and aromatic plants (MAPs) regarding their antioxidant and oxidative stress-modulating properties in cancer cell lines is presented.

## 2. Medicinal and Aromatic Plants

Medicinal and aromatic plants (MAPs) are vegetal materials that have pharmacological properties and also have aromatic and gastronomic uses [7]. The use of MAPs for drug preparation dates from approximately 5000 years ago [8], but nowadays, phytochemicals are involved in the formulation of medicines, food supplements, cosmetics, and other health-related products. In fact, approximately 50% of all drugs currently in clinical trials are derived from plants [9].

It is estimated that more than 80% of the world's medicinal plants grow in Asia and America [7, 10]. MAPs may exert several biological functions, for instance, extracts from MAPs could act as antimicrobial agents and reduce significantly the viability of pathogens of clinical and plant pathology interest. In the light of the increasing problem of antibiotic resistance, the antimicrobial properties of plant extracts may be of high value in clinical medicine. Some bioactive compounds have shown antifungal activity, which is also of interest in medicine, in pharmaceutics, and in plant sciences [11].

Another application of herbal products takes advantage of their high flavonoid content that confers them free radical scavenging properties that may, in turn, reduce cellular oxidative damage and ultimately help in the maintenance of intracellular antioxidant defenses [12]. In addition, bioactive molecules extracted from MAPs have revealed anti-inflammatory properties through TNF-*α* inhibition and *in vitro* decrease of nitric oxide generation [13]. Natural antioxidants might be more beneficial than synthetic ones, with the latter causing potential health side effects during long intake periods [14].

Since the discovery of the vinca alkaloids in the 1950s and their further application in cancer therapy, the interest in MAPs has increased [15]. The chemical components of many MAPs have been used in large extent for pharmaceutical studies. Bioactive compounds from plants had led to the discovery of several drugs with potential therapeutic value in cancer [16].

A number of plant-based anticancer drugs are currently under clinical studies [17] (see Table 1). Amin et al. [18] have classified plant-derived anticancer drugs into four classes according to their mechanisms of action: (1) methyl transferase inhibitors, (2) antioxidants, (3) histone deacetylase inhibitors (HDACi), and (4) mitotic disruptors. Methyl transferase inhibitors prevent cytosine methylation in CpG islands related to alterations in chromatin, causing gene silencing. The net effect is cell death via apoptosis [19]. The second class, antioxidants, can scavenge free radicals, preventing, therefore, ROS-related cellular and DNA damage. Inhibitors of histone deacetylases, the third group of plant-derived drugs act also as proapoptotic agents, by activating either the intrinsic or extrinsic pathway [20]. This type of inhibitors, however, might trigger cell death processes via necrosis and autophagy in some cell lines [21]. Finally, the fourth group, mitotic disruptors, cause damage to tubulin in the microtubules; hence, they prevent cell division and induce apoptosis [22].

## 3. Oxidative Stress

**Figure 1:**
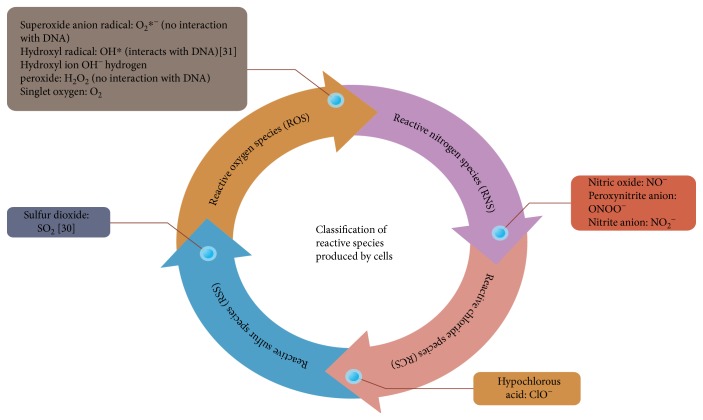
Classification of reactive species produced by cells [30, 31].

In eukaryotic cells, the most important redox process is aerobic respiration, which is mediated by mitochondrial electron transfer. This leads to the generation, in turn, of most of the endogenous intracellular reactive species (RS). For a proposed classification of RS that are produced within cells, see Figure 1 [29].

Generation of RS might be (1) exogenous, from sources such as UV radiation, toxic chemicals, cigarettes, drugs, pollutants, alcohol, physiological changes (aging, injury, and inflammation), and exercise or (2) endogenous, from intracellular sources including peroxisomes, mitochondrial metabolism, and the NADPH oxidase complex and myeloperoxidases, which play a fundamental role in maintaining a balanced cellular metabolism [32].

Oxidative stress (OS) is defined as an imbalance in ROS levels and cellular antioxidant endogenous mechanisms. Although ROS are involved in innate immunity and inflammatory signaling through pathogen elimination [32], ROS precise functions inside the complex metabolic network, however, are not clear [33].

High ROS concentrations have a major negative impact in biomolecules (nucleic acids, lipids, and proteins) [34], with high levels of ROS associated with atherosclerosis, cancer, diabetes, rheumatoid arthritis, cardiovascular diseases, chronic inflammation, stroke, and septic shock [35–38].

Intracellular ROS reduction is regulated by antioxidant enzymes such as glutathione peroxidases (GPx), catalases (CAT), and superoxide dismutases (SOD). However, in some cases, antioxidant defense mechanisms may be not enough to maintain a redox balance and might become easily saturated, causing permanent genome impairment and toxicity [39].

In cancerous cell lines, high levels of ROS are necessary to maintain a fast proliferation rate. Szatrowski and Nathan have reported that some cancer types such as melanoma, neuroblastoma, colon carcinoma, and ovarian carcinoma generate large quantities of hydrogen peroxide [40]. Furthermore, several oncogenes and tumor suppressors are affected by the presence of ROS [41]. Chemotherapy and radiation treatments increase intracellular ROS in order to eliminate cancer cells, but ROS production affects also surrounding normal cells, generating in turn damage to DNA and to several other biomolecules [42]. At the cellular level, ROS production effects are dose-dependent. A comprehensive understanding, however, of the complex ROS network, cytotoxicity pathways, and involved molecular metabolic mechanisms as a whole requires extensive analysis and experimentation.

**Figure 2:**
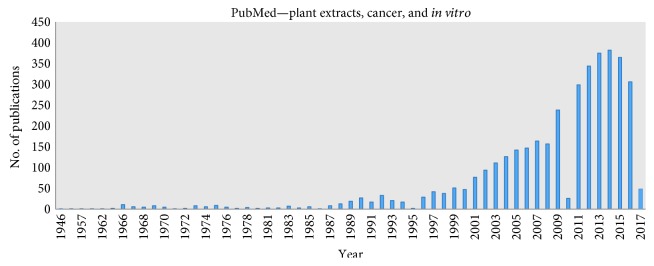
Number of publications related with plant extracts, cancer, and *in vitro*.

The number of publications, in a PubMed search for *in vitro* effects of plant extracts on cancer cell lines, has increased substantially over the last 10 years, as shown in Figure 2. This reflects on the potential value and interest in research regarding bioactive compounds from plants and cancer.

## 4. *In Vitro* Cell Line Models

Cell culture techniques provide a relatively easy and affordable way into eukaryotic experimentation. Cell culture is a very useful tool for the study of pathologic/normal mechanisms in cell biology and is considered essential when screening for potential new drugs for complex diseases, such as cancer. The use of a primary culture from certified vendors and proper culture media with adequate supplements and growth factors are crucial to secure consistent and stable *in vitro* cell functions. The use of immortalized cell lines facilitates i*n vitro* research, but normal cell lines need to be included as experimental controls [43].

Cell line models have been widely used in OS research and also in the study of new candidate molecules for the treatment of OS-related diseases. For instance, procarbazine, an azo derivative, has been part of cancer treatment schemes since its discovery 50 years ago. Procarbazine's mechanism of action as anticancer drug involves oxidation of the molecule and production of intracellular hydrogen peroxide [44].

Regulation of ROS is fundamental for homeostasis and normal functioning of cells. Several studies have addressed this complex series of phenomena. For instance, in a study by Schlenker et al., OS was evaluated using a bile duct epithelial cell (BDE) model to determine the earliest effects of RS in cell injury. The study authors found that OS causes a significant decrease in cell volume and affects multiple cell functions such as ion permeability, activation of ion channels, and apoptosis [45]. Glorieux et al. [39] compared the expression of catalase in two cell line models (250MK normal mammary epithelial primary cells and MCF-7 breast adenocarcinoma cells) and evaluated OS resistance to extended exposure to high hydrogen peroxide concentrations. The results showed overexpression of catalase in the MCF7 model, suggesting that adaptive responses to oxidative stress might be regulated by chromatin remodeling.

As shown in Figure 2, research on *in vitro* screening of MAP-derived antioxidants has been on the rise in the last years. In the next section, we will look at several promising applications of MAPs based on data from *in vitro* cancer studies.

**Figure 3:**
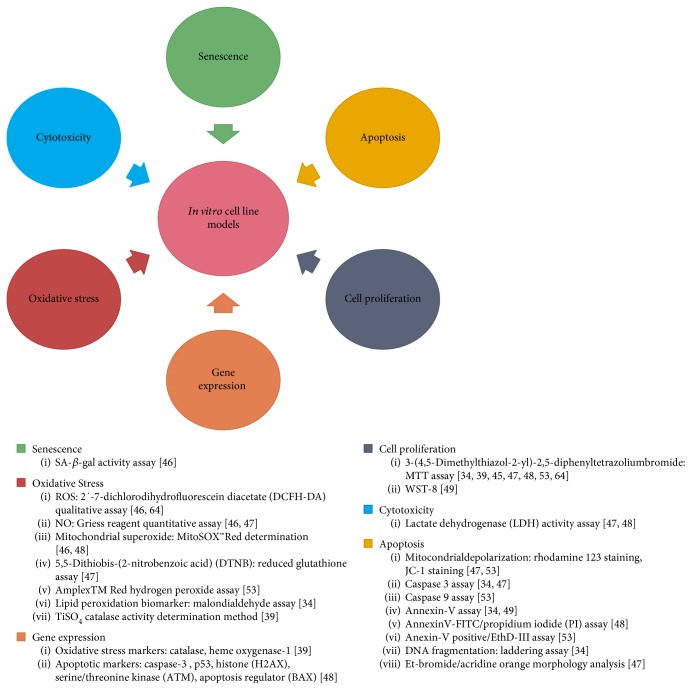
Most commonly used methods for MAP evaluation on *in vitro* cancer cell line models.

The methodology used in all the studies presented in this review has been summarized in Figure 3.

### 4.1. MAPs: New Anticancer Compounds

#### 4.1.1. Garlic

A study by Yedjou and Tchounwou evaluated the effect of garlic extract in an HL-60 model (human leukemia cells). This extract was found cytotoxic (via apoptosis) and an inductor of oxidative stress on tested cells. An interesting finding of the above study is the potential of malondialdehyde (MDA) as a biomarker of oxidative stress associated with caspase activation and DNA fragmentation. Mechanistic data regarding the observed cell responses to garlic extract is, however, still lacking [34].

#### 4.1.2. Apigenin

In a study on the *in vitro* effect of apigenin, a natural polyphenolic compound rich in flavones, on cell lines HT-29 (human colorectal adenocarcinoma) and HCT-15 (Dukes' type C-colorectal adenocarcinoma) models, the authors [46] showed that apigenin has significant antiproliferative and proapoptotic properties. Apigenin's mechanism of action includes reduction of mitochondrial membrane potential and production of free radical species, including mitochondrial superoxide. Apigenin is also a senescence inductor and thus is potentially suitable for cell-based models in aging research.

#### 4.1.3. Le Pana Guliya (LPG)

The anticancer properties of this herbal mixture used in traditional medicine have been assessed on several cancer cell lines (HepG2-hepatocellular carcinoma, HeLa-cervix adenocarcinoma, and CC1-normal rat fibroblasts) [47]. In the above work, the authors found that LPG had an intense antiproliferative effect on HepG2 and HeLa cells after 24 hours of exposure. LPG caused a reduction of protein synthesis in both cell lines in comparison to the normal CC1 line control. Glutathione (GSH) and nitric oxide (NO) measurements confirmed induction of oxidative stress, while LPG-mediated caspase activation and DNA fragmentation confirmed a proapoptotic effect of the mixture on the specific cell lines studied.

#### 4.1.4. Caffeic Acid (CA)

Chemotherapeutics are usually toxic for both noncancer and cancer cells. Appropriate combinations of available drugs are crucial to effectively kill cancer cells, reduce side effects and improve survival rates. A study by Tyszka-Czochara et al. [48] evaluated the individual and combinatory effects of synthetic metformin (Met) and the natural polyphenolic antioxidant caffeic acid (CA) on HTB-34 cells (cervical epidermoid carcinoma). The authors found that CA activates 5′-adenosine monophosphate-activated protein kinase (AMPK), involved in cell energy regulation. Met downregulates several enzymes (ACLY, FAS, ELOVL6, and SCD1). Although both agents have apoptotic effects in cancer cells, CA specifically increases ROS production, meanwhile Met inhibits fatty acid biosynthesis. When tested in combination, a significant antiproliferative net effect was shown.

#### 4.1.5. Bigelovin

The effect of this sesquiterpene lactone naturally produced by *Inula helianthus* aquatic on HT-29 and HCT 116 colorectal cancer cell lines was studied in a recent work by Li et al. [49]. The authors found a marked antiproliferative effect caused by apoptosis induction. The mechanism of action is a rise in ROS production in a process that involves several steps: (1) multi-caspase activation, (2) G2/M cell cycle arrest, and (3) DNA damage mediated by upregulation of death receptor 5 (DR5). These results support further research on a potential use of bigelovin in colorectal cancer therapeutics.

#### 4.1.6. Linalool

Iwasaki et al. in a recently published work [50] studied the anticancer properties of this monoterpenoid alcohol found in more than 200 plant species. The authors exposed HCT116 (colorectal carcinoma) and CCD-18Co (human colon fibroblast) cells to linalool and observed inhibition of cell proliferation by induction of apoptosis via hydroxyl radical-mediated OS. Interestingly, a lipid peroxidation marker for oxidative stress was also found. Results of the above work suggest that linalool may be a potentially valuable compound for novel chemotherapeutics due to its antiproliferative properties on targeted cancer cells.

#### 4.1.7. Curcumin

Curcumin is a bioactive compound isolated from *Curcuma longa* L. that has shown many pharmacological activities, such as modulation of the nuclear NF*-κ*B signaling pathway. Hong et al. [51] showed that curcumin nanosuspensions exert a dose-dependent cytotoxic effect on HeLa and HepG2 cell lines. Additionally, when comparing the nanosuspension with curcumin solution, curcumin nanoscale preparations showed to be more toxic presumably because nanostructures might enter the tumor via endocytosis. In another study by Banerjee et al., the synergistic effect of curcumin and docetaxel, a chemotherapeutic used in metastatic prostate cancer, was tested. Stronger cytotoxicity and proapoptotic effects, as well as reduced expression of CDK-1 and COX-2 were demonstrated when exposing human prostate cancer DU145 and PC3 cells to a combination of both agents [52].

### 4.2. MAPs: ROS Protective Agents in Cancer Research

The role of natural antioxidants in oxidative stress-related diseases has been studied for several years. MAPs can act as protective agents against OS and thus their potential for use in such diseases might be worth exploring.

A study of five antioxidant fractions from seaweed *Fucus spiralis* on H₂O₂-treated MCF-7 cells (breast adenocarcinoma) [53] showed a reduction of ROS production, induction of apoptosis through caspase 9 activation, and depolarization of the mitochondrial membrane. From the five antioxidant fractions studied in the above work, only the F4 fraction revealed biological significance showing cytoprotective properties against hydrogen peroxide toxicity, with an interesting potential use as oxidative stress modulator.

In another recent study, Giacoppo et al. [54] evaluated the effect of cannabigerol (CBG), from *Cannabis sativa,* on a hydrogen peroxide ROS-induced murine RAW 264.7 macrophage model. This study identified the selective receptor antagonists needed to elucidate CBG's known antioxidant properties at the mechanistic level. The authors found that activation of CB2 receptors is involved in CBG effects, with receptor inhibition leading to lower oxidation as a cytoprotective effect. CBG was found to downregulate several oxidative markers through inhibition of phosphorylation and modulation of MAPK. In addition, CBG exerted antioxidant activity by inducing superoxide dismutase-1 (SOD-1) overexpression, which causes an imbalance in proapoptotic factors to avoid cell death.

Plant compounds such as flavonoids, polysaccharides, and phytohormones could function as radioprotectors, meaning they can protect adjacent normal cells from the harmful effects of radiation therapy. It has been proposed that the above compounds might also offer protection against ionizing occupational and environmental radiation exposure [42]. In a recently published study, Szejk et al. [10] demonstrated that the pretreatment of human lymphocytes with some polyphenolic compounds isolated from plants from the Rosaceae/Asteraceae family could prevent ^(60)^Co *γ*-radiation DNA damage in exposed lymphocytes. Furthermore, these natural polyphenols could inhibit lipid peroxidation and activate antioxidant enzymes.

Conversely, plant products could act as radiosensitizers by enhancing the effect of ionizing radiation therapy against radioresistant cells. A study conducted by Wang et al. [55] showed that resveratrol, a well-known antioxidant compound, operate synergistically with ionizing radiation to induce autophagy, cause DNA damage, induce apoptosis, and inhibit DNA repair in a glioma stem cell line SU-2 model. In a study by Sayin et al. [56], resveratrol showed antioxidant activity either by ROS-scavenging or by intracellular antioxidant-enzyme induction in human normal cell lines (coronary artery endothelial cells (HCAEC)). The above study's data suggest that resveratrol could help prevent diseases related with the harmful and accumulative effect of ROS.

Lastly, it is important to mention that many studies currently in place evaluate the protective effect of novel antioxidant plant compounds against oxidative stress. Due to the complexity of biological systems, an interesting approach in antioxidant molecules testing might be the construction of an *in vitro* oxidative stress cell model. Hydrogen peroxide is a chemical that is frequently used for oxidative stress induction in cultured cells; however, concentrations and exposure times vary extensible and may require standardization to assure reproducibility and data comparability [56–58].

## 5. Conclusion

The use of *in vitro* cell models is critical for a comprehensive understanding of the biological properties of plant-derived compounds in cancer research. According to the European Union Reference Laboratory for Alternatives to Animal Testing (EURL-ECVAM), there are several *in vitro* cell models accepted for drug development applications. In the case of oxidative stress, studies on cell models help to integrate multiple metabolic routes and identify novel plant-based drugs or therapeutic targets. For instance, human-induced pluripotent stem cells (hiPSC) might be a valuable model for neurotoxicity studies with the use of the oxidative stress biomarker Nrf2 [59]. Such a model would also be an interesting addition to the currently available *in vitro* toxicology testing platforms.

Evaluation of potential natural drugs for modulation of oxidative stress in cancer might, in our opinion, be addressed efficiently and rigorously using cell line models. The NCI60 human tumor cell line anticancer drug screen panel had been used for testing of natural crude extracts collections since 1989. The panel requires previous individual cell line screening, which has led, for example, to the discovery of bortezomib, the first therapeutic proteasome inhibitor for the treatment of multiple myeloma [60].

Additionally, it is important to consider that a wide range of subtypes of cancer cell lines is currently available through certified vendors and collections. Colorectal cancer cell lines, for instance, show different genomic patterns and mRNA expression profiles [61] and may also show common mutations [62]. Therefore, the use of appropriately cultured cells with authenticated genetic and biochemical profiles, along with information on receptors, ion channels, and receptor signaling pathways are essential if reliable data is wanted from assays that assess the potential therapeutic properties of plant-derived biocompounds. The Cancer Cell Line Encyclopedia (CCLE) [63] offers robust preclinical information that integrates the genomic diversity of several human cancers and may facilitate new drug discovery strategies.

In conclusion, carefully planned experiments on cell culture models allow a throughout assessment of the molecular pathways involved in the mechanism of action of a bioactive compound. The use of these models for evaluation of ROS production and oxidative stress modulation properties of plant-derived biocompounds is well documented and growing. Consequently, *in vivo* experiments with animal models might be optimized based on *in vitro* data. Information from *in vitro* and *in vivo* studies will, in turn, facilitate and improve further clinical trials [49].

## 6. New Perspectives

Cell culturing aims at mimicking *in vivo* environment. Three-dimensional (3-D) cultures closely emulate tumor formation processes by synthetic tumor microenvironment mimics (STEMs). Models of human lung epithelial cells, pulmonary vascular endothelial cells, and human bone marrow-derived mesenchymal stem cells have been used for induction of ROS production and evaluation of upregulated efflux transporters [64]. Finally, new genome editing technologies such as CRISPR are currently in use for the study of molecular level responses to plant-derived bioactive compounds. Potential chemotherapeutic uses of these compounds for modulation of oxidative stress or ROS-mediated selective toxicity might, by this means, be explored using fast and affordable novel strategies.

## Figures and Tables

**Table 1:** Plant-derived natural products approved for cancer treatment.

Class	Isolated from	Mechanism of action	References
*Vinca alkaloids* (vinblastine, vincristine, vinorelbine, vinflunine)	*Catharanthus roseus* or *Vinca rosea*	Disruption of formation of the mitotic spindle; tubulin inhibitors; deregulation of actin cytoskeletons; microtubule destabilization and depolymerization; cell death via apoptosis.	[23, 24]
*Epipodophyllotoxins* (etoposide, teniposide, etoposide phosphate)	*Podophyllum peltatum* L.	Cell cycle arrest; DNA strand damage; cell death via apoptosis.	[25, 26]
Taxanes (paclitaxel, paclitaxel albumin-stabilized nanoparticle formulation, docetaxel, cabizitaxel)	*Taxus brevifolia* L.	Mitotic inhibitors; microtubule disruptors; apoptosis is induced through stabilization of microtubules.	[27]
Camptothecins (irinotecan and topotecan)	*Camptotheca acuminata*	Inhibition of the nuclear protein topoisomerase I.	[28]

## Abbreviations

^(60)^Co: Cobalt-60
ACLY: ATP citrate lyase
AMPK: 5′-Adenosine monophosphate-activated protein kinase
ATM: ATM serine/threonine kinase
BAX: Bcl-2-associated X protein
BDE: Bile duct epithelial cells
CA: Caffeic acid
CAT: Catalases
CB2: Cannabinoid receptor type 2
CBG: Cannabigerol
CC1: Normal rat fibroblast cell line
CCD-18Co: *Homo sapiens* colon normal cell line
CCLE: Cancer Cell Line Encyclopedia
CDK-1: Cyclin-dependent kinase 1
COX-2: Cyclooxygenase-2
CRISPR: Clustered regularly interspaced short palindromic repeats
DCFH-DA: Dichlorodihydrofluorescein diacetate
DNA: Deoxyribonucleic acid
DR: Death receptor
DTNB: 5,5-Dithiobis-(2-nitrobenzoic acid)
DU145: Human prostate cancer cell line
ELOVL6: ELOVL fatty acid elongase 6
EURL-ECVAM: European Union Reference Laboratory for Alternatives to Animal Testing
FAS: Fatty acid synthase
GPx: Glutathione peroxidases
GSH: Glutathione
H₂O₂: Hydrogen peroxide
HCAEC: Human coronary artery endothelial cells
HCT 116: Human colon carcinoma cell line
HCT-15: Dukes' type C-colorectal adenocarcinoma cell line
HDACi: Histone deacetylase inhibitors
HeLa: Cervix adenocarcinoma cell line
HepG2: Hepatocellular carcinoma cell line
hiPSC: Human-induced pluripotent stem cell
HL-60: Human promyelocytic leukemia cells
HT-29: Human colorectal adenocarcinoma cell line
HTB-34: Cervical epidermoid carcinoma cell line
LDH: Lactate dehydrogenase
LPG: Le Pana Guliya
MAPK: Mitogen-activated protein kinase
MAPs: Medicinal and aromatic plants
MCF-7: Human breast adenocarcinoma cell line
MDA: Malondialdehyde
Met: Metformin
mRNA: Messenger ribonucleic acid
MTT: 3-(4,5-Dimethylthiazol-2-yl)-2,5-diphenyltetrazolium bromide
NADPH: Nicotinamide adenine dinucleotide phosphate reduced
NCBI: National Center for Biotechnology Information
NCI60: The US National Cancer Institute (NCI*) 60* human tumour cell line anticancer drug screen
NF-*κ*B: Nuclear transcription factor-kappaB
NO: Nitric oxide
Nrf2: Nuclear factor erythroid 2-related factor 2
OS: Oxidative stress
PC3: Human Caucasian prostate adenocarcinoma cell line
PCR: Polymerase chain reaction
RAW 264.7: Macrophage; Abelson murine leukemia virus transformed from mouse cell line
ROS: Reactive oxygen species
RS: Reactive species
SA-*β*gal: Senescence-associated *β* galactosidase
SCD1: Stearoyl-CoA desaturase-1
SOD: Superoxide dismutases
STEMs: Synthetic tumor microenvironment mimics
SU-2: Glioma stem cell line
TiSO_4_: Titanium(II) sulfate
TNF-*α*: Tumor necrosis factor alpha
WST-8: Water soluble tetrazolium salt.
